# Epigallocatechin-3-Gallate Prevents the Acquisition of a Cancer Stem Cell Phenotype in Ovarian Cancer Tumorspheres through the Inhibition of Src/JAK/STAT3 Signaling

**DOI:** 10.3390/biomedicines11041000

**Published:** 2023-03-23

**Authors:** Sahily Rodriguez Torres, Loraine Gresseau, Meriem Benhamida, Yuniel Fernandez-Marrero, Borhane Annabi

**Affiliations:** 1 Laboratoire d’Oncologie Moléculaire, Département de Chimie, and CERMO-FC, Université du Québec à Montréal, Montreal, QC H3C 3J7, Canada; 2 Cell Biology Department, NuChem Sciences, Montreal, QC H4R 2N6, Canada

**Keywords:** EGCG, cancer stem cells, spheroids, ovarian cancer, STAT3

## Abstract

Three-dimensional tumorsphere cultures recapitulate the expression of several cancer stem cell (CSC) biomarkers and represent an effective in vitro platform to screen the anti-CSC properties of drugs. Whereas ovarian carcinoma is among the leading causes of death for women, ovarian CSC (OvCSC), a highly malignant subpopulation of ovarian cancer cells, is thought to be responsible for therapy resistance, metastasis, and tumor relapse. Epigallocatechin-3-gallate (EGCG), a diet-derived active polyphenol found in green tea leaves, can suppress ovarian cancer cell proliferation and induce apoptosis. However, its capacity to prevent the acquisition of cancer stemness traits in ovarian malignancies remains unclear. Here, we exploited the in vitro three-dimensional tumorsphere culture model to explore the capacity of EGCG to alter CSC biomarkers expression, signal transducing events and cell chemotaxis. Total RNA and protein lysates were isolated from human ES-2 ovarian cancer cell tumorspheres for gene assessment by RT-qPCR and protein expression by immunoblot. Real-time cell chemotaxis was assessed with xCELLigence. Compared with their parental adherent cells, tumorspheres expressed increased levels of the CSC markers *NANOG*, *SOX2*, *PROM1*, and *Fibronectin*. EGCG treatment reduced dose-dependently tumorspheres size and inhibited the transcriptional regulation of those genes. Src and JAK/STAT3 signaling pathways appeared to be relevant for CSC phenotype and chemotactic response. In conclusion, these data highlight and support the chemopreventive benefits of the diet-derived EGCG and its capacity to target intracellular transducing events that regulate the acquisition of an invasive CSC phenotype.

## 1. Introduction

Ovarian carcinoma is among the leading causes of death in women as its high mortality rate is, in part, the consequence of the lack of early symptoms, physical signs, and robust tumor biomarkers [1]. In addition, resistance to standard cancer therapies, including chemotherapy and radiotherapy, is thought to be responsible for ovarian cancer recurrence and metastasis [2,3]. This is due in part to cancer-initiating/cancer stem-like cells (CSC), which are defined as a small highly malignant subpopulation of cancer cells with greater tumor-initiating ability. Strategies to prevent the acquisition of cancer stemness, or to target ovarian CSC (OvCSC) to overcome therapy resistance in ovarian cancer, have recently led to innovative therapeutic approaches to prevent tumor relapse [4,5]. Among the most recent research avenues, epigenetic diet approaches against CSC are emerging as a very new strategy with a promising future for treating cancer patients [6,7,8].

CSC can be formed through oncogenic transformation of normal stem cells, but they can also be formed by de-differentiation of bulk tumour cells. Thus, factors boosting the expansion of normal stem cell pools or increasing the acquisition of stemness traits by tumour cells can have substantial repercussions on cancer origin and progression. The role of lifestyle factors, such as high caloric diet, alcohol drinking, and smoking, contribute to the widening of stem cell pools, and the induction of CSC features in tumors are also hypothesized [9]. Epigenetic modifications induced by bioactive dietary compounds are thought to be beneficial [10]. Many of these chemicals have anticancer effects and may help to avoid cancer. Several investigations have found that a variety of dietary substances have epi-genetic targets in cancer cells. Importantly, new research demonstrates that dietary treatments can change normal epigenetic states and reverse aberrant gene activation or silencing.

Naturally occurring substances, mostly phytochemicals, have received a great deal of attention in recent years due to their broad safety profile, capacity to target heterogeneous populations of cancer cells, and critical signalling pathways. Phenolic compounds represent such a vast group of substances with anticarcinogenic functions, anti-inflammatory, and anti-oxidative activities [11]. It appears that these characteristics may aim at neutralizing CSC development, their microenvironment, and metabolism in part through epigenetic mechanisms. Thus, targeting CSC and relevant signaling pathways by phytochemicals has recently been considered as a novel approach for breast cancer therapy [12].

Epigallocatechin-3-gallate (EGCG), a biological active polyphenol found in green tea leaves, can suppress ovarian cancer cell proliferation, and induce apoptosis [13], but its specific effects on stemness traits in ovarian malignancies remain unclear. It is therefor mandatory to explore the chemopreventive properties of EGCG targeting CSC proliferation and survival [14,15]. Polyphenols have also been linked to the prevention of cancer drug resistance [16]. EGCG has been shown to be a potent inhibitor of EOC cell growth, with most of its effects being mediated by apoptosis [17]. Rao and coworkers observed the drop in cell survival and DNA synthesis together with cell cycle arrest in the human ovarian cancer cell line SKOV-3 after treatment with EGCG, which was indicative of a pro-apoptotic cell state [18].

Here, we generated an in vitro ovarian cancer spheroid model from a primary culture of ES-2 ovarian cell carcinoma. Transcriptomic analysis confirmed the increased expression of classical CSC-associated genes promoting CSC-like characteristics in ovarian cancer cells. Among those genes are CSC biomarkers, cell cycle arrests molecules that contribute to maintain an undifferentiated and pluripotent state, while others are involved in cell motility, self-renewal, and chemoresistance. We also found induction of mesenchymal and epithelial genes characteristic of hybrid cell state that favors CSC metastatic spread. We further addressed the role of signaling pathways involving Src and JAK/STAT in tumorspheres in both the acquisition of a CSC phenotype and in the functional response to lysophosphatidic acid (LPA), a biolipid that stimulates ovarian tumor cell invasion and metastasis [19]. Our study now fills the knowledge gap that would help better ascertain the chemopreventive benefits of diet-derived polyphenols in general, with a specific capacity to target intracellular transducing and transcriptional events that regulate the acquisition of an invasive and chemoresistant CSC phenotype.

## 2. Materials and Methods

### 2.1. Materials

Sodium dodecyl sulfate (SDS) and bovine serum albumin (BSA) were purchased from Sigma-Aldrich Corp (St. Louis, MO, USA). Cell culture media was obtained from Life Technologies Corp (Carlsbad, CA, USA). Electrophoresis reagents were purchased from Bio-Rad Laboratories (Hercules, CA, USA). The HyGLO™ Chemiluminescent HRP (horseradish peroxidase) Antibody Detection Reagents were from Denville Scientific Inc. (Metuchen, NJ, USA). Micro bicinchoninic acid (BCA) protein assay reagents were from Pierce (Micro BCA™ Protein Assay Kit; Thermo Fisher Scientific, Waltham, MA, USA). The JAK family tyrosine kinase inhibitor AG490 was from Calbiochem (La Jolla, CA, USA). The monoclonal antibodies against GAPDH (D4C6R), pAKT (Ser473) (D9W9U, #12694), caspase-3, and the polyclonal antibodies against PARP (#9542), pSrc (Tyr416, #2101), STAT3 (79D7, #4904), and BCL-2 (50E3, #2870) were all from Cell Signaling Technologies (Danvers, MA, USA). The rabbit polyclonal antibody against CD133 (ab19898) was from Abcam (Toronto, ON, Canada). HRP-conjugated donkey anti-rabbit and anti-mouse immunoglobulin (Ig) G secondary antibodies were from Jackson ImmunoResearch Laboratories (West Grove, PA, USA). EGCG was from MP Biomedicals (Solon, OH, USA). All other reagents were from Sigma-Aldrich Corp.

### 2.2. Cell Culture

The American Type Culture Collection provided the human serous carcinoma-derived ES-2 ovarian cancer cells (ATCC, Manassas, VA, USA). McCoy’s 5a Modified Medium for ES-2 Cells (Wisent, 317-010-CL), containing 10% fetal bovine serum (Life Technologies, 12483-020), 100 U/mL penicillin, and 100 mg/mL streptomycin, was used to culture the cells in a monolayer (Wisent, 450-202-EL). Cells were grown at 37 °C in a humidified 95–5% (*v*/*v*) air-CO_2_ combination. Celprogen provided human ovarian cancer stem cells (OvCSC) (San Pedro, CA, USA). Cells were cultivated as monolayers at 37 °C in a humidified atmosphere (5% CO_2_) using the appropriate expansion and undifferentiation media, as well as matrix pre-coated flasks, according to the manufacturer’s recommendations (Celprogen).ES-2 tumorsphere formation was adapted from established protocols [20,21,22,23,24] and performed as follows: 80–90% adherent ES-2 monolayer cells were trypsinized and plated in non-adherent bacterial dishes at a density of 2 × 10^5^ cells/mL in complete media for 24 h. Following that, the supernatant was removed and serum-free McCoy’s 5a Modified Medium was supplemented with 10 ng/mL human basic fibroblast growth factor (Gibco, Thermo Fisher, 13256029), 20 ng/mL human epidermal growth factor (Gibco, Thermo Fisher, PHG0315), 5 μg/mL insulin (Sigma Aldrich, I3536), and bovine serum albumin (BSA) (Sigma Aldrich, A9418-5G) at 4% was carefully added to the dishes. Cells were maintained at 37 °C in a humidified atmosphere of 95% air and 5% CO_2_. Under an inverted phase-contrast microscope, the spheroid formation and growth were seen, and the size of the spheroids was determined from at least three different experiments.

### 2.3. Total RNA Isolation, cDNA Synthesis, and Real-Time Quantitative PCR

Total RNA was extracted from cell monolayers or tumorspheres using 1 mL of TriZol reagent for a maximum of 3 × 10^6^ cells, as the manufacturer recommends (Life Technologies, Gaithersburg, MD, USA). 1–2 μg of total RNA were reverse-transcribed for cDNA synthesis using a high-capacity cDNA reverse transcription kit (Applied Biosystems, Foster City, CA) or, in the case of the gene array, the R2 First Strand kit (QIAGEN, Valen-cia, CA, USA). Prior to PCR, the cDNA was kept at −80 °C. Real-time quantitative PCR was used to measure gene expression using iQ SYBR Green Supermix (Bio-Rad, Hercules, CA, USA). The Icycler iQ5 (Bio-Rad) was used for DNA amplification, and product identification was accomplished by detecting the binding of the fluorescent dye SYBR Green I to double-stranded DNA. The following primer sets were from QIAGEN: GAPDH (Hs_GAPDH_1_SG, QT00079247), Peptidylprolyl Isomerase A (PPIA) (Hs_PPIA_4_SG, QT01866137), β-Actin (Hs_Actb_2_SG, QT01680476), Snail (Hs_SNAI1_1_SG, QT00010010), Slug (SNAI2) (Hs_SNAI2_1_SG, QT00044128), Fibronectin (Hs_FN1_1_SGQT00038024), Prominin-1 (CD133) (Hs_PROM1_1_SG, QT00075586), and NANOG (Hs_NANOG_2_SG, QT01844808). The relative quantities of target gene mRNA were normalized against internal housekeeping genes PPIA and GAPDH. The RNA was measured by following a ∆C_T_ method employing an amplification plot (fluorescence signal vs. cycle number). The difference (∆C_T_) between the mean values in the triplicate samples of target gene and the housekeeping genes was calculated with the CFX manager Software version 2.1 (Bio-Rad) and the relative quantified value (RQV) was expressed as 2^−∆CT^.

### 2.4. Human Apoptosis and Cancer Stem Cell PCR Arrays

The RT^2^ Profiler^TM^ PCR arrays for Human Apoptosis (PAHS-012ZD) and Human Cancer Stem Cells (PAHS-176ZD) were used according to the manufacturer’s protocol (QIAGEN). The detailed list of the key genes assessed can be found on the manufacturer’s website (https://geneglobe.qiagen.com/us/product-groups/rt2-profiler-pcr-arrays; accessed on 13 January 2022). Using real-time quantitative PCR, we reliably analyzed the expression of a focused panel of genes related to the inflammatory response, including some of the cancer-associated adipocytes markers already published. Relative gene expression was calculated using the 2^−∆∆CT^ method (“delta-delta” method), in which C_T_ indicates the fractional cycle number where the fluorescent signal crosses the background threshold. This method normalizes the ∆C_T_ value of each sample using five housekeeping genes (*B2M*, *HPRT1*, *RPL13A*, and *GAPDH*). The normalized FC values were then presented as average FC = 2 (average ^∆∆C^ T). To minimize false positive results, only genes amplified less than 35 cycles were analyzed. The resulting raw data were then analyzed using the PCR Array Data Analysis Template (http://www.sabiosciences.com/pcrarraydataanalysis.php; accessed on 5 June 2022). This integrated web-based software package automatically performs all ∆∆C_T_-based FC calculations from the uploaded raw thresholded cycle data.

### 2.5. Western Blot

After lysis of the cells in a solution containing 1 mM NaF and Na_3_VO_4_, proteins (10–20 μg) were separated by SDS-polyacrylamide gel electrophoresis (PAGE). Proteins were then electro-transferred to polyvinylidene difluoride membranes and blocked for 1 h at room temperature in Tris-buffered saline (150 mM NaCl, 20 mM Tris-HCl, pH 7.5) with 0.3% Tween-20 (TBST; Bioshop, TWN510-500). Membranes were washed in TBST and incubated overnight with the appropriate primary antibodies (1/1000 dilution) in TBST containing 3% BSA and 0.1% sodium azide (Sigma-Aldrich) at 4 °C and in a shaker. After three washes with TBST, the membranes were incubated 1 h with horseradish peroxidase-conjugated anti-rabbit or anti-mouse IgG at 1/2500 dilutions in TBST containing 5% nonfat dry milk. Immunoreactive material was visualized by ECL.

### 2.6. Chemotactic Cell Migration Assay

The xCELLigence system’s Real-Time Cell Analyzer (RTCA) Dual-Plate (DP) Instrument was used to conduct cell migration studies (Roche Diagnostics). Trypsinization and seeding of adherent cell monolayers or tumorspheres (30,000 cells/well) onto CIM-Plates 16 (Roche Diagnostics) was performed. These migration plates contain gold electrode arrays on the bottom side of the membrane in place of the standard Transwells (8 μm pore size) to allow for real-time cell migration measurement. The bottom side of the chamber’s membrane was covered with 25 μL of 0.15% gelatin in PBS and incubated for an hour at 37 °C prior to cell seeding. Chemotaxis was monitored for 8 h using LPA as chemoattractant in the presence or not of EGCG. The impedance values were measured by the RTCA DP Instrument software and were expressed in arbitrary units as Normalized Cell Migration Index. Each experiment was performed three times in duplicate.

### 2.7. Statistical Data Analysis

Unless otherwise stated, data and error bars were expressed as the mean standard error of the mean (SEM) of three or more separate experiments. The Kruskal–Wallis test was used to assess hypotheses, followed by a Mann–Whitney test or a Dunn–Tukey post-test (for data with more than three groups) (two group comparisons). Probability values of less than 0.05 or 0.01 were judged significant and indicated in the figures as (*) or (**), respectively. The statistical analysis software GraphPad Prism 7 was used for all calculations (San Diego, CA, USA).

## 3. Results

### 3.1. Epigallocatechin-3-Gallate Inhibits ES-2 Ovarian Clear Cell Carcinoma Tumorsphere Formation

**Figure 1:**
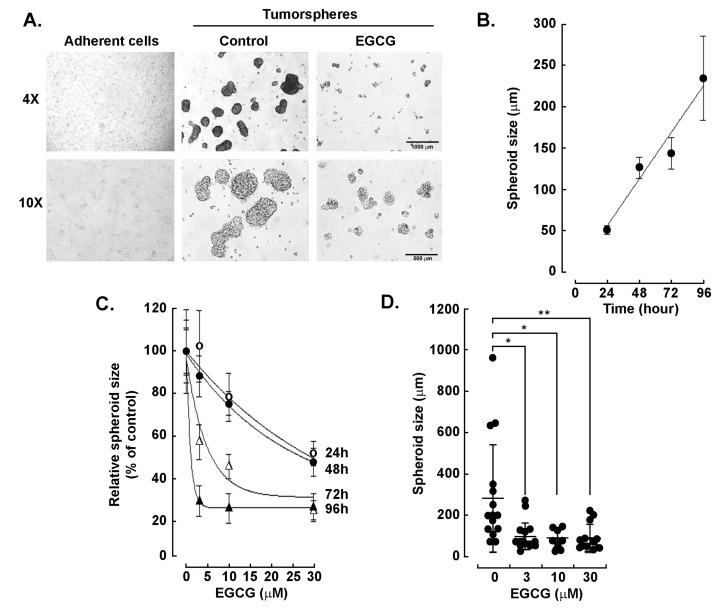
Epigallocatechin-3-gallate inhibits ES-2 ovarian clear cell carcinoma tumorsphere formation. (**A**) Tumorspheres were generated from adherent human ES-2 ovarian cancer cell monolayer cultures, as described in the Methods section, in the absence or presence of 30 μM EGCG. Representative phase contrast pictures were taken at 96 h at 4× (upper panels) and 10× (lower panels) magnification. The spheroid formation and growth were monitored under an inverted phase-contrast microscope, and the size of the spheroids was measured from at least three independent experiments. Representative tumorsphere images are shown. (**B**) Relative spheroid perimeter was measured at the indicated time, and tumorsphere growth kinetic assessed for up to 96 h. (**C**) Tumorspheres growth was performed for the indicated times and in the presence of increasing EGCG concentrations. (**D**) Statistical analysis of tumorspheres growth at 96 h in the presence of increasing EGCG concentrations. All experiments were performed in triplicate and repeated three times, data were analyzed using one-way analysis of variance, and the results were expressed as mean ± SD (* *p* < 0.05; ** *p* < 0.01).

Tumorspheres formation was first assessed starting from adherent human ES-2 ovarian cancer cell monolayer cultures, as described in the Methods section in the absence or presence of 30 μM EGCG. Representative phase contrast pictures were taken at 96 h at a 4× (Figure 1A, upper panels) and 10× (Figure 1A, lower panels) magnification. It is clearly apparent that the impact of EGCG against tumorspheres was to prevent their formation. Relative tumorspheres size increased with time for up to 96 h (Figure 1B). Tumorspheres growth was also performed for 24–96 h and was found to dose-dependently decrease in the presence of increasing EGCG concentrations (Figure 1C). Statistical analysis of tumorspheres growth at 96 h found the impact of EGCG at 3, 10, and 30 μM (Figure 1D) significant. Collectively, this validates the tumorspheres culture protocol. Whether EGCG, besides altering tumorspheres growth, also affected any CSC phenotype, was next assessed.

### 3.2. Ovarian Cancer Tumorspheres Acquire a Cancer Stem Cell Molecular Signature

**Figure 2:**
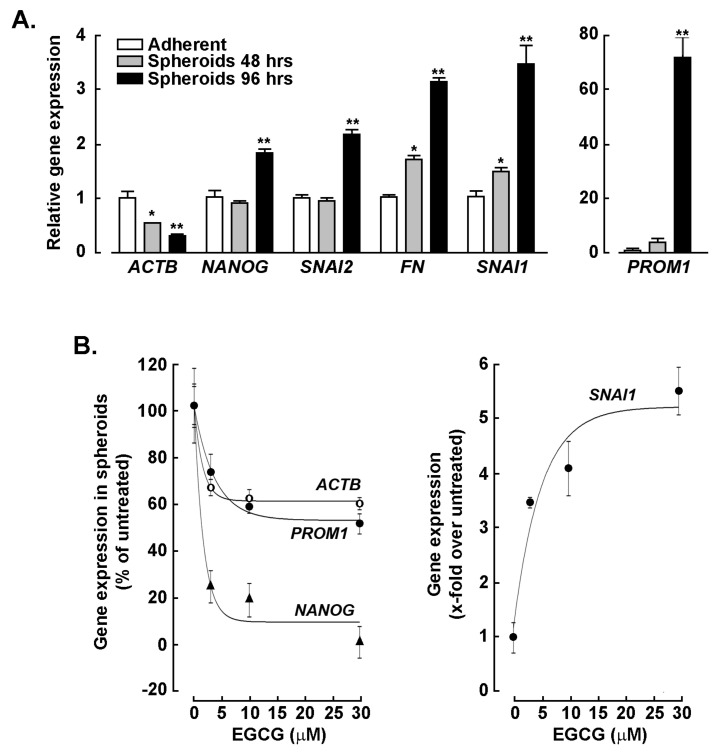
Ovarian cancer tumorspheres formation correlates with increased cancer stem cell biomarkers expression. (**A**) Tumorspheres were generated from adherent human ES-2 ovarian cancer cell monolayer cultures as described in the Methods section for 0 (adherent monolayer cells), 48 (grey bars), and 96 (black bars) hours. Total RNA was extracted, and RT-qPCR was performed to assess the gene expression levels of *β-Actin* (ACTB), *NANOG*, *SNAI2*, *Fibronectin* (FN), *SNAI1*, and *PROM1*. (**B**) Gene expression in adherent cells (t = 0; untreated), and in tumorspheres treated for 96 h in the presence of increasing EGCG concentrations, was performed by RT-qPCR. All experiments were performed in triplicate and repeated three times. Data were analyzed using one-way analysis of variance and the results were expressed as mean ± SD (* *p* < 0.05; ** *p* < 0.01).

Tumorspheres were generated from adherent human ES-2 ovarian cancer cell monolayer cultures as described in the Methods section. Total RNA was extracted, and RT-qPCR was performed to find decreased gene expression levels of *β-Actin* (ACTB) as reported elsewhere [25], but increased *NANOG*, *SNAI2*, *Fibronectin* (FN), *SNAI1* (Figure 2A, left panel), and *PROM1* (CD133) (Figure 2B, right panel) in tumorspheres formed at 48 (grey bars) and 96 (black bars) hours. While the induced gene expression of *NANOG* and *PROM1* decreased dose-dependently with EGCG, that of *β-Actin* further decreased in tumorspheres treated for 96 h (Figure 2B, left panel). As a contrast, *SNAI1* gene expression was upregulated by EGCG (Figure 2B, right panel).

### 3.3. EGCG Transcriptional Regulation of the Human ES-2 Ovarian Cancer Stem Cell Molecular Signature in Tumorspheres

**Figure 3:**
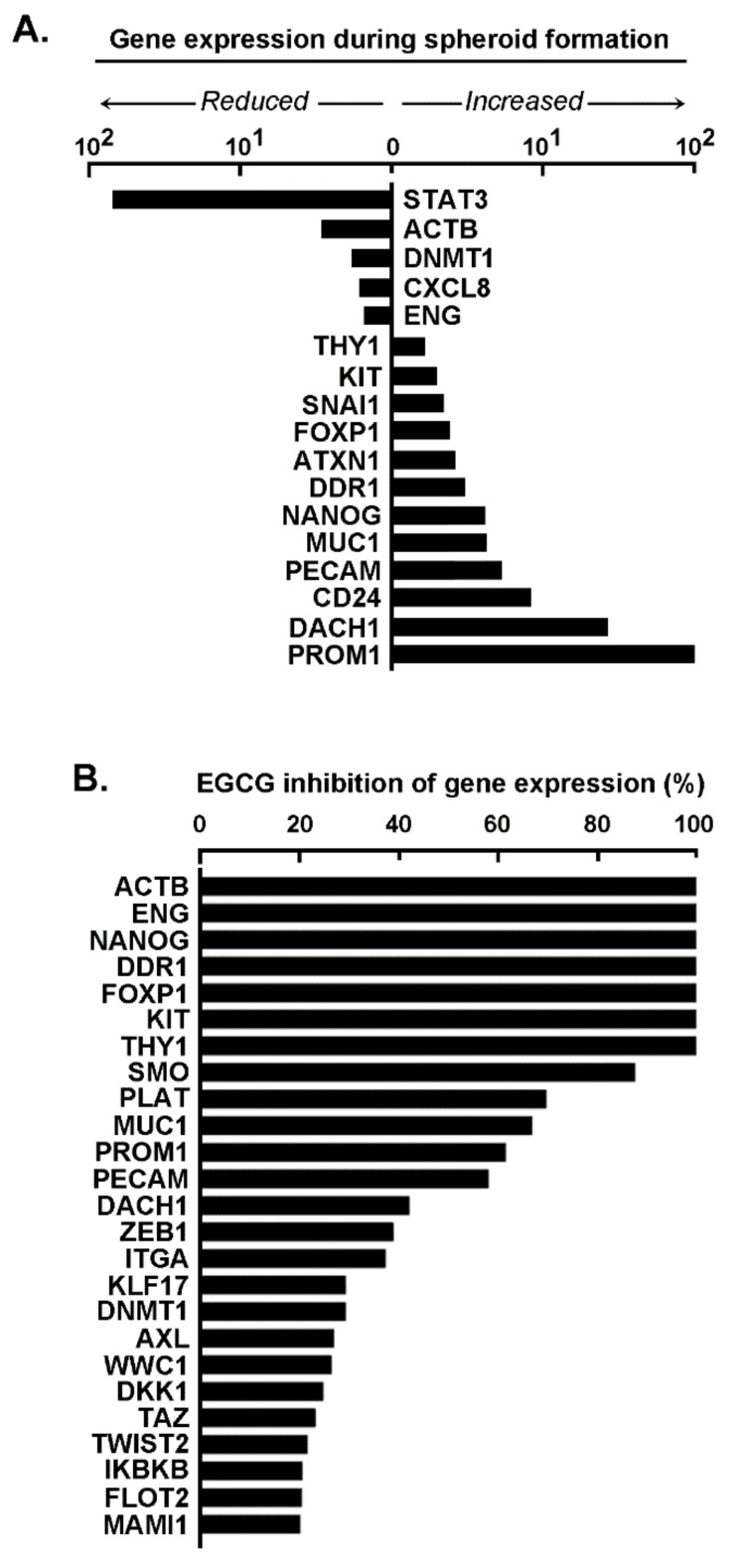
Transcriptional validation of the human ES-2 ovarian cancer stem cell phenotype and impact of EGCG. Tumorspheres were generated from adherent human ES-2 ovarian cancer cell monolayer cultures, as described in the Methods section, in the absence or presence of 30 μM EGCG. Total RNA was extracted from either adherent monolayers (t = 0 h) or tumorspheres at 96 h, and RT-qPCR was performed using the RT2-Profiler gene array to assess the expression levels of cancer stem cell-associated genes. (**A**) Ratios of spheroid gene expression over adherent cells were performed and expressed on a logarithmic scale in untreated cells. (**B**) Ratios of tumorspheres grown in the presence of 30 μM EGCG were calculated, and the extent of EGCG inhibition was presented as a percentage.

Tumorspheres were generated from adherent human ES-2 ovarian cancer cell monolayer cultures as described in the Methods section in the absence or presence of 30 μM EGCG. Total RNA was extracted from either adherent monolayers or tumorspheres formed at 96 h, and RT-qPCR performed using the RT2-Profiler gene array to assess the expression levels of cancer stem cell-associated genes. Gene expression ratios were obtained by comparing tumorspheres over adherent cells and were expressed on a logarithmic scale in untreated cells (Figure 3A; increased), and confirmed the inductions of *PROM1*, *NANOG*, and *SNAI1*, as well as other markers including *THY1*, *CD24*, *KIT*, *FOXP1*, and *DACH1*. On the other hand, *β-Actin* and other markers, including *ENG*, *CXCL8*, *DNMT1*, and *STAT3*, were downregulated upon spheroids formation (Figure 3A; reduced). The impact of EGCG on the CSC molecular signature of tumorspheres was also assessed and expressed as extent of gene inhibition. EGCG was found to efficiently inhibit, from 20–100%, most of the induced genes involved in tumorspheres formation, including *CD133* and *NANOG* (Figure 3B). This confirms that the acquisition of a CSC phenotype can be altered by EGCG during tumorspheres formation, and that such regulation occurs at the transcriptional level.

### 3.4. EGCG Induces a Pro-Apoptotic Phenotype in Ovarian Cancer Tumorspheres

**Figure 4:**
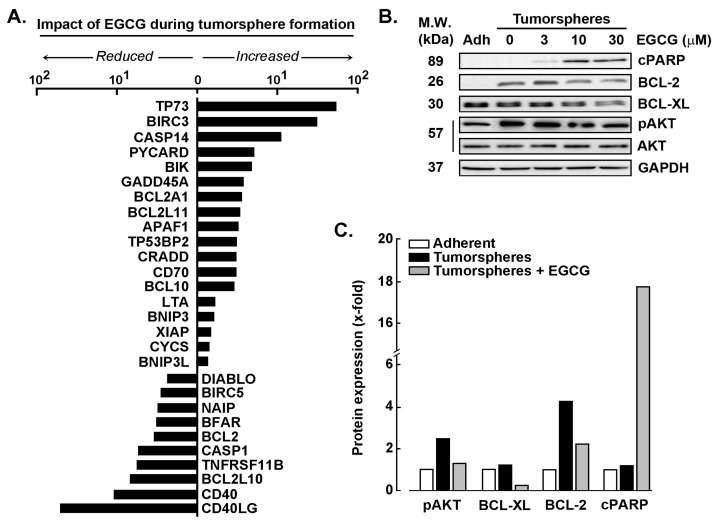
EGCG induces a pro-apoptotic phenotype in ovarian cancer tumorspheres. Tumorspheres were generated from adherent human ES-2 ovarian cancer cell monolayer cultures as described in the Methods section in the absence or presence of 30 μM EGCG. (**A**) Total RNA was extracted from tumorspheres at 96 h, and RT-qPCR performed using the RT2-Profiler gene array to assess the expression levels of apoptosis-associated genes. (**B**) Cell lysates were also isolated for protein expression levels, and immunoblotting of BCL-2, BCL-XL, pAKT, AKT, and cPARP (30 μg of protein/well). (**C**) Representative densitometry analysis of BCL-XL, BCL-2, pAKT, and cPARP protein expression expressed in x-fold inductions over basal adherent monolayer cells (white bars), untreated tumorspheres (black bars), and tumorspheres grown in the presence of 30 μM EGCG (grey bars).

The pro-apoptotic impact of EGCG was next assessed on tumorspheres. Cancer cells frequently overexpress proteins that play an important role in resisting the activation of the apoptotic cascade, named anti-apoptotic proteins. We found that expression levels of the anti-apoptotic BCL-2 and prosurvival pAKT were higher in the ovarian CSC spheroids compared to their adherent parental condition. This result indicates that anti-apoptotic pathways are operating in ovarian CSC spheroids, which may contribute to the maintenance of a resistance phenotype. Total RNA was extracted from tumorspheres generated upon 96 h in the presence of EGCG, and RT-qPCR was performed using the RT2-Profiler gene array to assess the expression levels of apoptosis-associated genes. Several pro-apoptotic genes were found increased and this included, among others, *TP73*, *BIRC3*, and *APASF1* (Figure 4A, increased). On the other hand, some genes were downregulated, and these included anti-apoptotic *CD40LG*, *BCL2L10*, and *BCL2* (Figure 4A, decreased). When the impact of EGCG was assessed at the protein level in cell lysates (Figure 4B), the expression of the anti-apoptotic BCL-2 and of the prosurvival pAKT was found to be increased upon tumorsphere formation (Figure 4C), but not that of BCL-XL. When tumorspheres were formed in the presence of increasing EGCG concentrations, the expression of BCL-2, BCL-XL and phosphorylation of AKT decreased, and this was accompanied by increased pro-apoptotic cleaved PARP (cPARP) expression (Figure 4C). BCL-2 has an oncogenic role because its overexpression increases AKT activity, which in turn plays a central role in inhibiting apoptosis in a variety of tumor types [26]. Constitutive activation of AKT (pAKT) has been observed in several human cancers, including ovarian, lung, breast, and prostate, and is associated with increased cancer cell proliferation and survival.

### 3.5. Pharmacological Inhibition of the Src Signaling Pathway Alters the Acquisition of a Cancer Stem Cell Phenotype in Ovarian Cancer Tumorspheres

**Figure 5:**
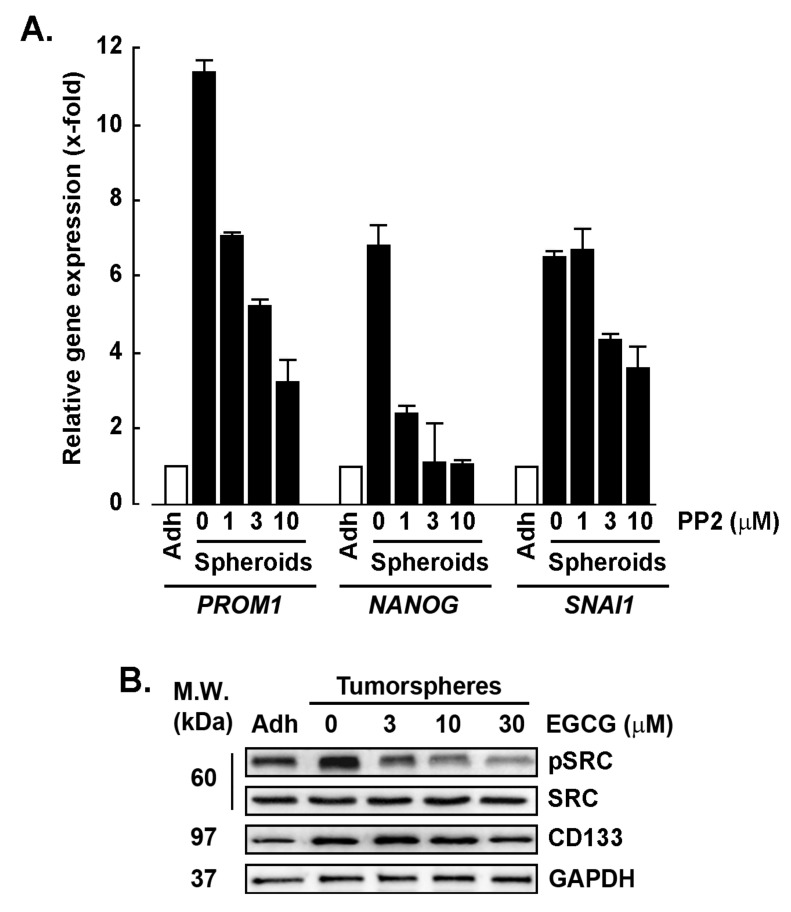
Pharmacological inhibition of the Src signaling pathway alters the acquisition of a stem cell phenotype in ovarian cancer tumorspheres. Tumorspheres were generated from adherent human ES-2 ovarian cancer cell monolayer cultures as described in the Methods section in the absence or presence of (**A**) increasing concentrations of either the Src inhibitor PP2 or (**B**) EGCG. RT-qPCR was performed to assess the gene expression levels of *PROM1*, *NANOG*, and *SNAI1*. Protein expression levels of pSrc and CD133 were assessed in adherent monolayers and in tumorspheres by immunoblotting.

The contribution of the Src signaling pathway was explored through the pharmacological inhibition strategies of its phosphorylated state. First, the reversible and ATP-competitive Src family kinases inhibitor PP2 was found to dose-dependently prevent the tumorspheres-induced transcript levels of CSC markers *PROM1*, *NANOG*, and *SNAI1* (Figure 5A). At the protein level, EGCG was found to mimic PP2 inhibition of Src phosphorylation effects, and this concomitantly prevented tumorspheres-induced CD133 protein expression (Figure 5B). This suggests that a signaling axis requiring Src activation is involved in the acquisition of a CSC phenotype upon tumorsphere formation. Given that EGCG was previously documented to alter the Src/Janus kinase (JAK)/STAT3 pathway [27,28], as well as EMT in glioblastoma [20], the contribution of STAT3 was next assessed.

### 3.6. STAT3 Regulates the Acquisition of a Cancer Stem Cell Phenotype and Chemotactic Response of Ovarian Cancer Tumorspheres to Lysophosphatidic Acid

**Figure 6:**
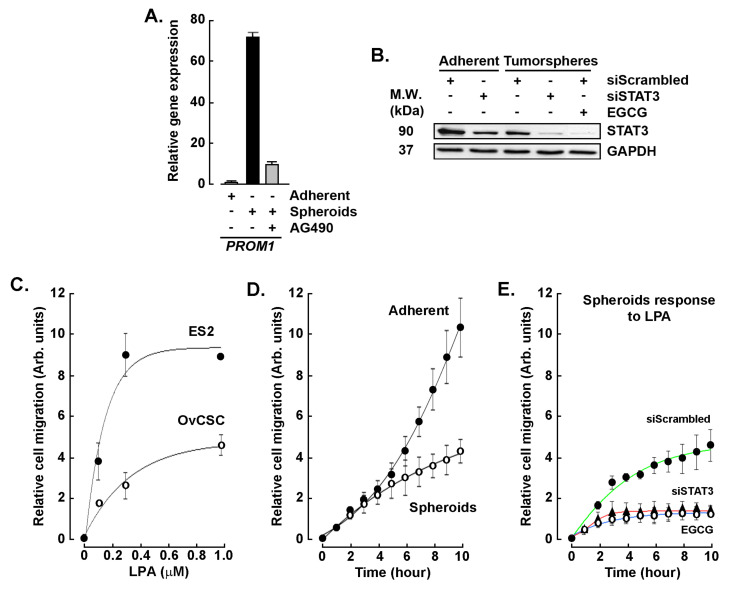
STAT3 regulates the chemotactic response of ovarian cancer tumorspheres to lysophosphatidic acid. Transient gene silencing of STAT3 (siSTAT3) was performed in adherent ES-2 ovarian cancer cell monolayers as described in the Methods section. Control cells were transfected with a siRNA scrambled sequence. Tumorspheres were next generated as described in the Methods section in the absence or presence of 30 μM EGCG. (**A**) *PROM1* gene expression was assessed by RT-qPCR in either ES-2 monolayers, and in spheroids generated in the presence or not of the JAK/STAT3 inhibitor AG490. (**B**) Cell lysates were isolated and levels of STAT3 and GAPDH proteins were assessed by Western blotting from the siScrambled- or siSTAT3-transfected cells. Tumorspheres were also generated in the presence of 30 μM EGCG. (**C**) Real-time cell migration was performed to assess ES-2 monolayer cells or commercially available OvCSC chemotactic response to increasing concentrations of Lysophosphatidic Acid (LPA). (**D**) Adherent and tumorsphere cell migration was assessed in time in response to 1 μM LPA. (**E**) Real-time cell migration of tumorspheres where STAT3 was silenced (siSTAT3) or not (siScrambled) was assessed as described in the Methods section in response to LPA and in the presence or absence of 30 μM EGCG.

The JAK/STAT3 signaling pathway was further explored here because *STAT3* transcript levels were among the genes significantly decreased upon tumorsphere formation and CSC phenotype acquisition (Figure 3A). We questioned whether the remaining basal levels of STAT3 activity might contribute to the CSC phenotype acquisition. Accordingly, use of the pharmacological JAK/STAT3 inhibitor AG490 prevented the induction of PROM1 expression upon tumorsphere formation (Figure 6A). Transient gene silencing of STAT3 was performed using siRNA to assess the overall functional chemotactic response of cells. STAT3 reduction upon spheroid formation was validated at the protein level, whereas silencing efficiency of STAT3 was also confirmed (Figure 6B). Interestingly, EGCG was also found to further decrease the levels of STAT3 in tumorspheres reaching levels equivalent to those obtained upon siSTAT3 (Figure 6B). The global role of STAT3 in the acquisition of a CSC phenotype in ES2 tumorsphere or in a commercially available ovarian cancer-derived CSC (OvCSC) model was further explored in terms of functional chemotactic response to the bioactive JAK/STAT3 inducer lysophosphatidic acid (LPA) [19]. It was found that LPA triggered a dose-responsive chemotactic effect, which was observed in both the ES-2 parental monolayer cultures as well as in OvCSC, although to a lesser extent (Figure 6C). When tumorspheres were generated and exposed to LPA, spheroids appeared to also respond less in time to an extent, similar to that observed in OvCSC (Figure 6D). Finally, silencing of STAT3 in tumorspheres was found to alter the chemotactic response to LPA, and this was efficiently mimicked by EGCG, suggesting that STAT3 displayed a crucial role in spheroids’ chemotactic response (Figure 6E).

## 4. Discussion

Cancers are heterogeneous tissues, and a layer of heterogeneity is determined by the presence of cells showing stemness traits, known as CSC. Evidence indicates that CSC are important players in tumor development, progression, and relapse. In ovarian CSC, an increased expression of the aldehyde dehydrogenase (ALDH) enzyme is, in fact, an essential mechanism that maintains drug-resistance [29], greater sphere-forming ability, and tumorigenesis [30]. Among the CSC biomarkers explored here in ovarian cancer tumorspheres, CD133 is of the most consistent markers of gynecological CSC [31,32]. While its biological functions remain elusive, CD133 is found to be overexpressed in tumor-initiating cells in several solid tumors, including melanoma, brain, colon, liver, lung, pancreatic, prostate, and ovarian cancers [33,34]. Accordingly, ovarian cancer cell spheroids could recapitulate an ALDH+/CD133+ phenotype in vitro and form tumors in vivo [35].

In addition to CD133, the stem cell transcription factor NANOG was also found induced in our ovarian tumorspheres in accordance with previous reports where it regulates cell proliferation and apoptosis [36]. NANOG has been found as overexpressed in many types of human cancers, including the head and neck, liver, lung, kidney, oral cavity, pancreas, prostate, ovary, and other organs [37]. An increase in gene and protein levels of NANOG in ovarian cancer cells was associated with higher sphere-forming capacities and drug and apoptosis resistance our results [38,39]. NANOG depletion reduced ovarian cancer cell proliferation, invasion, and stem-like characteristics [40]. NANOG further appears to regulate CSC populations through the induction of stemness surface markers CD133, CD44, EpCAM, and CD90 [36]. Of interest, NANOG expression correlated positively with levels of total and phosphorylated STAT3, suggesting a role for NANOG-mediated EMT and drug-resistance through the activation of the STAT3 pathway in epithelial ovarian cancer [41].

Other transcripts that were upregulated during ovarian cancer spheroids formation include DACH1, the Discoidin domain receptor (DDR1), the winged helix transcription factor Forkhead box P1 (FOXP1), and MUC1 (Figure 3A). Of specific interest, DDR1 is a collagen-activated receptor tyrosine kinase highly expressed in all histological subtypes of epithelial ovarian cancer compared with the normal ovarian surface epithelium [42] and has been ascribed a role in the JAK2/STAT3 pathway in sustaining pluripotency factors and self-renewal capabilities of metastatic CSC [43]. DDR1 overexpression in our ovarian spheroids model may contribute to the intrinsic chemoresistant phenotype supporting CSC traits since, similarly to the inhibitory effects of EGCG on DDR1, DDR1 knockdown significantly increased the sensitivity of ovarian cancer cell lines to cisplatin treatment, resulting in elevated apoptosis [44]. FOXP1 functions as an oncogene in epithelial ovarian cancer cells by promoting the CSC-like characteristics, while its overexpression led to an up-regulated expression of ABCG2, OCT4, NANOG, and SOX2 genes and protected cells against apoptotic cell death [45]. As we found that FOXP1 upregulation in ovarian cancer tumorsphere was significantly prevented by EGCG, FOXP1 may constitute an attractive target for the development of therapeutics to eliminate CSC in ovarian cancer [46]. Finally, MUC1 is a highly glycosylated type I transmembrane glycoprotein that is overexpressed in more than 90% of EOCs, including platinum-resistant tumors [47]. MUC1 also has an active role in apoptosis-resistant mechanisms and is associated with the induction of the EMT program in CSC [48]. A hybrid epithelial/mesenchymal phenotype has been observed in ovarian cancer associated with increased cancer cell stemness, poor survival, and resistance to therapy [49]. Tumor cells with hybrid epithelial/mesenchymal phenotypes have multiple advantages over cells that completed EMT, as hybrid cells are anoikis resistant, an essential trait for efficient metastasis [50].

THY1, CD24, and KIT (CD117) were also found induced in tumorspheres. THY1 expression is indicative of poor outcomes and is found to be higher in ovarian CSC than in non-CSC and promotes proliferation in ovarian cancer [51]. CD24 is linked to an increased metastatic and invasiveness potential in ovarian tumors and a shortened patient survival and is associated with signaling factors, such as Src kinase in lipid rafts microdomains, and requires STAT3 [52]. KIT (CD117)+ ovarian cancer cells manifest a striking higher tumorigenic activity than CD117-negative cancer cells and were able to generate the original tumor heterogeneity, suggesting self-renewal and multi-linage differentiation capabilities of these cells [53].

On the other hand, ovarian cancer tumorsphere formation was also reflected by decreased expression of DNMT1. DNA methylation status was directly regulated by DNMTs which possess de novo methylation activity. In hepatocellular carcinoma, DNMT1 downregulation resulted in significant demethylation of the *PROM1* promoter, resulting in its enhanced expression in a mechanism dependent on TGF-β stimulation [54]. EGCG’s capacity to further alter DNMT1 functions may translate into further lowering of the methylation level of the CG5 site in the NANOG promoter [55]. Epigenetic regulation through the inhibition of DNMT1 as a mechanism to alter stemness traits is a finding that is not yet reported for ovarian cancer cells.

Evidence supports the effects of EGCG targeting nasopharyngeal CSC-like capabilities in spheroid formation, self-renewal, and EMT signatures in TW01 and TW06 nasopharyngeal cancer cells [56]. This was thought to be mediated through the suppression of STAT3 signaling pathway and its downstream genes *BCL2*, *c-MYC*, and *Survivin*, which affect tumor growth by inducing apoptosis [57]. We found that another mechanism operated by EGCG to target ovarian cancer tumorspheres was therefore the induction of an apoptotic state. EGCG was able to suppress protein expression levels of BCL-2 and pAKT, and was able to induce cPARP in a dose-dependent manner (Figure 4B). In human endometrial cancer cells, EGCG treatment resulted in the suppression of anti-apoptotic protein BCL-2, the upregulation of pro-apoptotic BAX, and the activation of caspase-3 and PARP [58]. Multiple evidence already supports the induction of apoptosis by EGCG in ovarian cancer cells, but we provide, to the best of our knowledge, the first evidence of EGCG targeting ovarian tumorspheres with CSC phenotype through the induction of apoptosis.

The last objective of this work was to explore the role of signaling intermediates involved in the acquisition of a CSC phenotype upon ovarian cancer tumorsphere formation. Due to our prior work, we decided to focus on the role of the STAT3 pathway and its upstream-related protein Src in ovarian CSC spheroids and the effects of EGCG targeting these pathways. STAT3 is activated by several cytokines like IL-6 and IL-10 and growth factors, including EGF, FGF, and IGF. The binding of these molecules to their cognate receptors activates receptor-associated kinases like Janus kinases (JAKs) or non-receptor kinases like Src that phosphorylate STAT3 [59]. Once activated, STAT3 forms homodimers and translocates into the nucleus where it binds to the promotor region of target genes encoding BCL-2, c-Myc, cyclin D1, Survivin, MMP-2, and MMP-9, which promote tumorigenesis [60].

Src is a signal-transducing non-receptor protein kinase that plays a central role in the control of cell growth and differentiation, in part as an upstream activator of the STAT3 pathway. Overexpression and activation of Src family kinases have been identified in a range of human cancers [61]. Src is also involved in ovarian cancer development and in the maintenance of the ovarian CSC phenotype. Accordingly, Src has been overexpressed and activated in most of the late-stage ovarian tumors [62]. The inhibition of Src enhanced the cytotoxicity of cisplatin and paclitaxel in drug-sensitive ovarian cancer cells and restores sensitivity in drug resistant cells, and these effects are dependent on caspase-3 activity [63]. To test the relevance of the Src pathway in the acquisition of a CSC phenotype, we generated ovarian cancer tumorspheres in the presence of PP2, a Src inhibitor which suppressed the expression of two master regulators of the CSC phenotype, CD133 and NANOG. Evidence supporting that Src blockade targets CSC subpopulation was highlighted as a dual MEK and Src inhibitor decreased the ALDH1+ population, and reduced sphere-forming and tumor-initiating cells in tumor xenografts [64].

Finally, the CSC biomarker CD24 can affect Src activity and the subsequent STAT3 phosphorylation, pointing out the close link between stemness and Src/STAT3 molecular pathway [65]. We also found that the inhibition of Src reduced the transcriptional expression of Snail, indicating that this pathway is also involved in promoting EMT traits of the ovarian CSC spheroids. In line with this result, constitutive active MEK and Src led to sustained EMT in epithelial ovarian cancer cells [66]. An interesting result was that EGCG was able to suppress the expression of pSrc in a dose-dependent manner, pointing out that this could be one of its target molecules in the inhibition of the ovarian CSC phenotype. The addition of EGCG inhibited the expression of STAT3, and this corresponds with the suppression of CD133 protein levels.

## 5. Conclusions

In conclusion, while three-dimensional spheroids assays are commonly used to uncover more relevant ovarian tumor biology than classical culture conditions, one must acknowledge that such assays at this stage may still preclude direct clinical application. Interestingly, our study supports the impact of EGCG as a prospective targeting of CSC and in the potential prevention of metastasis in a pre-clinical in vivo study of chemically-induced mammary cancer in rats [67]. Here, our work highlights and supports the chemopreventive benefits of the diet-derived EGCG and its capacity to target intracellular transducing events that regulate the acquisition of an invasive ovarian CSC phenotype. More importantly, reducing drug efflux processes are among the mechanisms by which polyphenols such as EGCG increase the sensitivity of cancer cells to chemotherapeutic agents [16]. Future studies will therefore require defining their role in overcoming the chemoresistance phenotype of CSC.

## Data Availability

All data generated or analyzed during this study are included in this published article.

## Abbreviations

ACTB: β-Actin; CSC, Cancer stem cells; OvCSC; Ovarian CSC; EGCG, Epigallocatechion-3-gallate; LPA, Lysophosphatidic acid; EMT, Epithelial-to-mesenchymal transition; FN, Fibronectin; ALDH, Aldehyde dehydrogenase; JAK, Janus kinase.
